# Health system responsiveness in maternity care at Hadiya zone public hospitals in Southern Ethiopia: Users’ perspectives

**DOI:** 10.1371/journal.pone.0258092

**Published:** 2021-10-14

**Authors:** Ritbano Ahmed Abdo, Hassen Mosa Halil, Biruk Assefa Kebede, Abebe Alemu Anshebo, Minychil Demelash Ayalew, Selamu Abose Nedamo, Shamill Eanga Helill

**Affiliations:** 1 Department of Midwifery, College of Medicine and Health Sciences, Wachemo University, Hossana, Ethiopia; 2 Department of Anesthesia, College of Medicine and Health Sciences, Wachemo University, Hossana, Ethiopia; Public Library of Science, UNITED KINGDOM

## Abstract

**Background:**

Health system responsiveness refers to non-financial, non-clinical qualities of care that reflect respect for human dignity and interpersonal aspects of the care process. The non-clinical aspects of the health system are therefore essential to the provision of services to patients. Therefore, the main purpose of this study was to assess the responsiveness in maternity care, domain performance and factors associated with responsiveness in maternity care in the Hadiya Zone public Hospitals in Southern Ethiopia.

**Methods:**

A hospital-based cross-sectional study was employed on 413 participants using a systematic sampling technique from 1 July to 1 August 2020. An exit interviewer–administered questionnaire was used to collect data. EpiData (version 3.1) and SPSS (version 24) software were used for data entry and analysis, respectively. Bivariate and multivariable logistic regression were computed to identify the associated factors of health system responsiveness in maternity care at 95% CI.

**Results:**

The findings indicated that 53.0% of users gave high ratings for responsiveness in delivery care. In the multivariable logistic regression analysis, mothers aged ≥ 35 (AOR = 0.4; 95% CI = 0.1–0.9), urban resident (AOR = 2.5; 95% CI = 1.5–4.8), obstetrics complications during the current pregnancy (AOR = 2.1; 95% CI = 1.1–3.0), and caesarean delivery (AOR = 0.4; 95% CI = 0.2–0.7) were factors associated with poor ratings for responsiveness in maternity care.

**Conclusion:**

In the hospitals under investigation, responsiveness in maternity care was found to be good. The findings of this study suggest that the ministry of health and regional health bureau needs to pay attention to health system responsiveness as an indicator of the quality of maternity care.

## Introduction

The World Health Organisation (WHO) defines a maternal death as the death of a woman while pregnant or within 42 days of the termination of a pregnancy, irrespective of the duration and site of the pregnancy. The WHO further defines a maternal death as one that may be from any cause related to or aggravated by the pregnancy or its management, except unintentional or incidental causes. Maternal death is divided into two groups, direct and indirect obstetric deaths, which are based on the cause of death. Direct obstetric deaths are those “resulting from obstetric complications of the pregnant state and from interventions, omissions, incorrect treatment, or from a chain of events resulting from any of the above” [1]. Direct obstetric deaths account for nearly 75% of all maternal deaths [1, 2]. Indirect obstetric deaths are those maternal deaths “resulting from previous existing disease or disease that developed during pregnancy and not due to direct obstetric causes but were aggravated by the physiologic effects of pregnancy” [1].

Between 2000 and 2017, the maternal mortality rate dropped by about 38% worldwide [3]. However, it is still unacceptably high. Worldwide, approximately 295,000 women died during and following pregnancy and childbirth in 2017, and 94% of these deaths occurred in low-resource (low and lower middle-income) countries), with sub-Saharan Africa (SSA) accounting for nearly two thirds of the deaths. In Ethiopia, an estimated 14,000 maternal deaths occurred in 2017, yielding an overall maternal mortality ratio of 401 maternal deaths per 100,000 live births [3].

The government of Ethiopia has made maternal health a priority in its political agenda and has maintained its commitment to improving the health and survival of women in the country [4, 5]. With the aim of reducing maternal mortality to 267 deaths per 100,000 live births, a set of high-impact interventions have been implemented, including antenatal care, skilled birth services and postnatal care. Access to and utilization of key health care services through Health Extension Workers, the government’s flagship Programme, has been improved. Moreover, there has been an expansion of primary and secondary level health care through the accelerated expansion of health centres and hospitals throughout the country. In addition, the country has equipped a large proportion of these facilities with basic equipment and supplies and has staffed them with a trained health care workforce. Furthermore, health care finance (total health expenditure per capita) has improved significantly in the last decade due to the implementation of new schemes such as revenue retention and utilisation in health facilities; the opening of private wings in hospitals; community-based health insurance, and social health insurances. The health care delivery system has been augmented by the private and non-governmental organisation (NGO) sectors. Women’s participation in leadership and political structures has also shown significant improvements [4–6].

Meanwhile, health facilities have demonstrated an ever-increasing emphasis on meeting citizens’ expectations, improving responsiveness to patients and increasing both population and patient satisfaction [7]. Increasing health service quality is thought to increase compliance with medical treatment and to improve information transfer and utilisation of health services [8–10]. An important method to measure service quality is the concept of "responsiveness", which was introduced by the WHO in the World Health Report 2000 to measure service quality in an internationally comparable way [11]. Responsiveness refers to the clients’ universally legitimate expectations and measures the performance of the health system in terms of the extent to which they provide services as a response to their client’s needs, as well as the environments in which they are served [12]. It refers to non-financial, non-clinical qualities of care that reflect respect for human dignity and interpersonal facets of the care process [13–15]. The non-clinical aspects of the health system are therefore essential to the provision of services to patients [11, 15–17].

Even though considerable work has been done by the government to reduce maternal mortality arising from pregnancy-related complications, a high proportion of maternal deaths due to pregnancy-related complications has been reported in Ethiopia [18–21]. There is either limited evidence or a complete absence of evidence in a study area identifying the non-clinical aspects of the quality of maternity care. An understanding of this issue is an important step towards the implementation of interventions that would improve delivery care in Ethiopia. Therefore, the main purpose of this study was to assess the responsiveness in maternity care, domain performance and factors associated with responsiveness in maternity care in the Hadiya Zone public Hospitals in Southern Ethiopia.

## Methods and materials

A hospital-based cross-sectional study was conducted from 1 July to 1 August 2020 among Hadiya Zone public hospitals in Ethiopia. Hadiya Zone is one of the 14 zones in the Southern Nations, Nationalities, and Peoples’ Region, located southwest of Ethiopia and 233 km away from Addis Ababa. According to the 2019/20 Hadiya Zone Health Department report, the zone houses a total population of 1,688,820, of which 851,841 (50.44%) are females and 263,963 (15.63%) are urban inhabitants. The zone covers an estimated area of 3,542.66 km^2^. The zone has 4 hospitals (1 teaching and 3 primary hospitals), 61 health centres (2 private and 59 public), more than 162 private clinics and 311 health posts. According to the number of annual registrations at each hospital in 2011, all the hospitals served approximately 10,774 mothers per year on average [22].

The source population consisted of all mothers who had given birth in Hadiya zone Hospitals during the study period, and the study population consisted of randomly sampled mothers from this group. Mothers who had given birth in the hospitals during the study period were included, and women with mental illness and who were unable to hear and talk were excluded from the study because it was considered that they would not be able to provide the necessary information. A sample size of 422 was calculated using the one-sample population proportion formula assuming 50% as the responsiveness rate (there was no local data available on the topic) [23], 95% as the confidence interval (CI), 5% as the margin of error and 10% as the allowance for the non-response rate. Systematic sampling was employed, and all local hospitals were included (i.e. Wachemo University Nigist Eleni Mohammed Memorial Teaching Hospital [WUNEMMTH], Homecho Primary Hospital, Shone Primary Hospital and Gimbichu Primary Hospital). The study participants were allocated according to the proportion of patient inflow in each hospital. The k-value (k = 2) was estimated by dividing the total number of deliveries in the previous month by the sample size. The first mother was chosen by lottery from one of two deliveries on the first day of delivery in each hospital. One participant was selected from every two deliveries until the needed sample size was attained in each hospital. With only nine mothers declining to participate in the study, we had an overall response rate of 97.8% **(S1 Fig).**

The data were collected using a pre-tested structured exit interviewer questionnaire. The research questionnaire was developed based on the instruments used in the WHO’s multi-country research on health systems responsiveness questionnaires [11, 14] and the ReproQ [24] (**S1 File)**. The questionnaires were planned to collect information on socio-demographic characteristics (8 items) and eight components (domains) of responsiveness [25, 26]. Each domain involved assessing dignity (5 items), autonomy (3 items), confidentiality (3 items), communication (5 items), prompt attention (3 items), social support (3 items), choice and continuity (3 items) and basic amenities (5 items). Thus, these eight domains were measured with 3–5 items in each domain. The data were collected by 12 midwives with bachelor’s degrees under the supervision of 3 midwives with master’s degrees. All the midwives worked independently (i.e. outside the hospitals).

The questionnaire was initially prepared in English, translated first into the local language and then translated back into English by experts to check consistency. The questionnaire was pretested on 5% (21) of the sample size at Worabe Comprehensive hospital, and necessary modifications were made for the local context before data collection. Additionally, the data collectors and supervisors were trained for a day by the investigators of this study on the content of the questionnaire and the manner of data collection. All of the completed questionnaires were checked daily for completeness, accuracy, clarity, and consistency by the supervisors and principal investigators. Furthermore, the completeness and consistency of the variables during data entry and analysis were confirmed using frequency distributions. Cronbach’s alpha was used to assess the reliability of the items. The alpha coefficient of the overall responsiveness was 0.88. The alpha coefficients of communication, quality of basic amenities, confidentiality, dignity, prompt attention, autonomy, choice and social support domains were 0.85, 0.81, 0.80, 0.79, 0.75, 0.72, 0.71 and 0.70, respectively. All the coefficients were thus higher than the minimum acceptable level of 0.7.

### Study variables and measurements

#### Study variables

The dependent variable in this study is **responsiveness performance in maternity care.** Conversely, the independent variables include the following:

#### Sociodemographic factors

- Residence (rural or urban)
- Age of mother (≤20, 20–34 or ≥35 years)
- Religion (Protestant, Orthodox, Muslim or Catholic)
- Marital status (single, married, divorced or widowed)
- Ethnicity (Hadiya, Kambata, Silte, Gurage or Amhara)
- Occupation (housewife, employed, merchant, day labourer or student)
- Education (no formal education, primary or secondary education, preparatory school or college and higher education)

#### Obstetric characteristics

- Parity (primipara, multipara or grand multipara)
- Mode of delivery (vaginal or caesarean section)
- Current birth attendant
- Duration of labour (≤12 hours or >12 hours)
- History of abortion (yes/no based on the mother’s self-report)
- ANC follow up (<4 or ≥4 visits based on mother’s self-report)
- Obstetric complications during pregnancy
- Length of hospital stay
- Onset of labour (spontaneous, induced or elected caesarean)
- Maternal hospital admission during the antenatal or postnatal period (yes/no)
- Receiving an intervention (yes/no, instrumental delivery or caesarean section)
- Adverse child outcome

#### Responsiveness performance in maternity care

In this study, responsiveness performance in maternity care was studied using eight domains, namely, respect for a person’s dignity, autonomy to participate in health-related decisions, confidentiality, prompt attention, adequate quality of care, communication, access to social support networks, and choice of health care providers [25, 26]. First, sum scores per domain were calculated and transformed onto a scale of 1–10, to allow comparison between domains with a different number of items. Second, the total mean score was calculated. Finally, a score ≥ sample mean value was considered good responsiveness performance in maternity care. However, a score < sample mean value was regarded as poor responsiveness in maternity care. **Performance of domain (respect for person and client orientation domain):** Two responsiveness outcome measures were estimated to describe performance, namely question measures and domain measures. For the question measures, the five options answers were grouped into binary categories (‘good’ and ‘poor’). The ‘poor’ rating was used when a respondent reported the item as’ strongly disagree, ‘disagree’ or ‘moderately agree’ while the ‘good’ rating was used when a respondent reported the item as’ strongly agree or ‘agree’. Good was given a score of "1", and poor was given a score of "0". For the domain measures, if over 33% of the items were rated as’ poor ‘within a domain, the rating of’ poor ‘was used for each domain [25].

#### Obstetrics complications during pregnancy

Such as antepartum hemorrhage, hypertension disorders during pregnancy, polyhydramnios, chorioamnionitis, malpresentation, malposition, prolonged labour or/and others (present = 1 or absent = 0). **Adverse birth outcome**: a mother who gave as low birth weight, preterm, congenital malformation or/and stillbirth, and was classified as: “Yes” or “No”. **Parity** is defined as the number of times that she has given birth to a fetus with a gestational age of 28 weeks or more, regardless of whether the child was born alive or was stillborn. It is divided into three categories: Primipara, Multipara, and Grand Multipara. Primipara refers to a woman who has only had one child. A woman who has had two or more pregnancies is known as a multipara. Grand multipara: The fact of having given birth to more than four children.

### Data analysis

The data were checked and entered into EpiData version 3.1 and exported to the SPSS Version 24 statistical software for analysis. Descriptive analysis was used to describe the frequency distribution of each variable. The outcome variables were coded as “1” for poor responsiveness in maternity care whereas “0” for good responsiveness in maternity care **(S2 File)**. The association between the outcome variables (i.e., poor responsiveness in maternity care and independent variables) was analyzed using a logistic regression model [27, 28]. Initially, bivariate logistic regression analysis was performed on all independent variables. Multivariable logistic regression was then performed on variables that had a p-value ≤ 0.25 in the bivariate logistic regression analysis to assess the strength of the relationship between an outcome and several independent variables and to control for potential confounders. The degree of association between independent and dependent variables was assessed using an adjusted odds ratio with a 95% confidence interval. The P–value *<* 0.05 was considered as statistically significant in the multivariable model. Hosmer and Lemeshow’s goodness-of-fit test was used to assess whether the necessary assumptions were fulfilled.

### Ethics approval and consent to participation

Ethical clearance was secured from the Ethics Committee of the College of Medicine and Health Sciences, Wachemo University. Additionally, a permission letter was obtained from the hospitals authorities before commencing the data collection. The participants were informed about the purposes, procedures, potential risks and benefits of the study. Thereafter, written informed consent was obtained from each study participant. Informed consent was obtained from a parent or guardian for study participants younger than 18 years of age. Confidentiality was maintained throughout the study by excluding personal identifiers, such as names and addresses.

## Results

### Description of study participants

Four hundred and thirteen mothers participated in the study. Of the participants, 300 (72.6%), 61 (14.8%), 31 (7.5%) and 21 (5.1%) were from Nigist Eleni Muhammed Referral Hospital, Shone, Homecho and Gimbichu hospitals, respectively. Three hundred and twenty (77.5%) mothers were aged 20–34 years with a mean age (± SD) of 27.39 ± 5.31 years. The majority of the mothers, i.e. 391 (94.7%) were married, 310 (62.5%) were Hadiya ethnic, 295 (71.4%) were Protestants, 258 (62.5%) were housewives and 293 (70.9%) were urban residents. In terms of education, 20.8% had completed higher education, 30.5% had a secondary or preparatory level of education, and 27.8% had a primary level of education while 20.8% had no formal education.

Of the 413 study participants, 126 (30.05%) were primigravida and 19.1% were grand multipara. Fifty-two (17.5%) participants had ever experienced abortion, and 75 (18.2%) had faced obstetrics complications during the current pregnancy. The majority of the mothers, 375 in total (90.9%), had attended antenatal care follow up visits, while 157 (48.5%) had ≥ ANC4+ visits. The results of other socio-demographic factors and items related to obstetrics characteristics are indicated in **Table 1**.

**Table 1:** Socio-demographic and obstetric characteristics of mothers who had given birth in the Hadiya zone public hospitals, July 2020 (n = 413).

Characteristics	Category	Frequency	Percent
Age	≤20 years	36	8.7
	20–34 years	320	77.5
	≥35 years	57	13.8
Marital status	Single	14	3.4
	Married	391	94.7
	Divorced	3	.7
	Widowed	5	1.2
Religion	Protestant	295	71.4
	Orthodox	77	18.6
	Muslim	35	8.5
	Catholic	6	1.5
Ethnicity	Hadiya	310	75.1
	Kambata	37	9.0
	Silte	19	4.6
	Gurage	28	6.8
	Amhara	19	4.6
Occupation of mothers	Housewife	258	62.5
	Employee	80	19.4
	Merchant	42	10.2
	Daily laborer	13	3.1
	Students	20	4.8
Parity	Primipara	126	30.5
	Multipara	208	50.4
	Grand multipara	79	19.1
Onset of labour	Spontaneous	347	84.0
	Induction	52	12.6
	Elective caesarean delivery	14	3.4
Mode of delivery	Vaginal	347	84.0
	Caesarian delivery	66	16.0
Intervention*	No	328	79.4
	Yes, no emergency intervention	28	6.8
	Yes, emergency intervention	57	13.8
Duration of labour(n = 399)	≤12 hours	306	76.7
	>12 hours	93	23.3
Length of hospital stay	≤6hours	17	4.1
	6–24 hours	293	70.9
	> 24 hours	103	24.9
Adverse birth outcome **	No	340	82.3
	Yes	73	17.7
Hospital admission of mothers	No	364	88.1
	Yes	49	11.9

* Caesarean section or instrumental delivery.

**Adverse outcome based on self-reported asphyxia, congenital anomaly, infection, low birth weight, premature birth.

### Responsiveness performance in the maternity care

Two hundred and nineteen (53.0%) of the mothers in this study rated overall responsiveness performance during their maternity care as good, while 194 (47.0%) mothers reported responsiveness performance as poor. The performance of responsiveness in maternity care varied greatly across the domains studied. In maternity care, responsiveness performance was reported as good for the dignity domain (77%) and the choice and continuity domain (41.2%). Good performance ratings were achieved for the respect for person domain (70%) when compared to the client orientation domain (27.8%). The proportions of maternity care responsiveness domains are provided in **Table 2**.

**Table 2:** Client reported responsiveness for each, respect, orientation and over all domain in the public maternity care, July 2020.

Domain	Items	Responsiveness in maternity care			
		Good		Poor	
		n	(%)	n	(%)
Dignity	Treated with respect	348	84.3	65	15.7
	Respect privacy in physical examination	346	83.8	67	16.2
	Encouraged to ask questions	320	77.5	93	22.5
	Gave personal attention	340	82.3	73	17.7
	Free to discuss concerns	336	81.4	77	18.6
Domain responsiveness		318	77.0	95	23.0
Autonomy	Involved making decisions	354	85.7	59	14.3
	Received information about other types of treatments	284	68.8	129	31.2
	choice to refuse examinations or treatments	246	59.5	82	19.9
	Asked for permission before starting testing	331	80.1	103	24.9
Domain responsiveness		239	57.9	174	42.1
Confidentiality	Given the opportunity to speak privately	224	54.2	189	45.8
	Confidentiality of patient information	238	57.6	157	38.0
	Confidentiality of medical records	256	62.0	157	38.0
Domain responsiveness		251	60.8	162	39.2
Communication	Explained clearly	309	74.8	109	26.4
	Encouraged to ask questions	311	75.3	102	24.7
	Given enough time to ask questions	306	74.1	107	25.9
	Providers were responsive to my questions	319	77.2	94	22.8
	Providers listened carefully	328	79.4	85	20.6
Domain responsiveness		295	71.4	118	28.6
Respect for persons		289	70.0	124	30.0
Prompt attention	Received prompt attention	302	73.1	111	26.9
	Reasonable waiting time	307	74.3	94	22.8
	Reasonable traveling time to this health	311	75.3	102	24.7
Domain responsiveness		296	71.7	117	28.3
Social support	Allow visitors	157	38	256	62.0
	Allowed attendant stay	188	45.4	225	54.5
	Family and friends were able to bring foods	279	67.6	134	32.4
Domain responsiveness		186	45.0	227	55.0
Choice of providers	Choice of provider at health care unit	70	16.9	343	83.1
	Choice between units	199	48.2	214	51.8
	Provided by one health care provider	148	35.8	231	55.9
Domain responsiveness		172	41.6	241	58.4
Basic amenities	Cleanliness of toilets, examination room and linen	191	46.2	222	53.8
	Adequacy of space	266	64.4	147	35.6
	Cleanliness care provider hands and clothes	241	58.4	172	41.6
	Cleanliness of department, bedroom, and bathroom	235	56.9	178	43.1
	Waiting areas and rooms had good air quality	290	70.2	123	29.8
Domain responsiveness		211	51.1	202	48.9
Client orientation		115	27.8	298	72.2
Over all Responsiveness in maternity care		219	53.0	194	47.0

#### Factors associated with poor responsiveness performance in maternity care

A bivariate logistic regression analysis, as presented in **Table 3**, revealed the factors associated with the poor responsiveness performance reported in delivery care. These were urban residence, caesarean delivery, higher maternal education level, hospital admission of mothers, obstetrics complications during the current pregnancy, and students. Multivariable logistic regression found that a maternal age of ≥ 35, urban residence, obstetrics complications during the current pregnancy, students, and caesarean delivery were factors associated with the poor ratings for responsiveness performance in delivery care. Mothers living in urban areas were more likely to report poor responsiveness in delivery care (AOR = 2.5; 95% CI = 1.5–4.8). Similarly, mothers who had obstetric complications during the current pregnancy were 2.7 times more likely to report poor responsiveness in the delivery care than mothers with obstetrics complications during the current pregnancy (AOR = 2.7; 95% CI = 1.1–3.0). Mothers in the ≥ 35 years age group were 60% less likely to report poor responsiveness in the delivery care compared to mothers aged <20 years (AOR = 0.4; 95% CI = 0.1–0.9). Furthermore, mothers who had a vaginal delivery were 60% less likely to report poor responsiveness in the maternity care compared to those who had undergone a caesarean delivery (AOR = 0.4; 95% CI = 0.2–0.7).

**Table 3:** Factors associated with poor responsiveness in maternity care of Hadiya zone public hospitals, July 2020.

Character	Category	Responsiveness in maternity care		OR at 95% Cl	
		Good	Poor	Crude OR	Adjusted OR
Residence	Rural(ref.)	82	38	1	1
	Urban	137	156	2.6(1.6, 3.8)	2.5(1.5, 4.8)**
Mode of delivery	Vaginal	175	172	0.5 (0.3, 0.9)	0.4(0.2, 0.7)**
	Caesarian delivery(ref.)	44	22	1	.1
Hospital admission of new–borns	No(ref.)	199	165	1	**1**
	Yes	20	29	1.9(1.1, 4.0)	1.8(0.9, 3.4)
Obstetric complications	No (ref.)	187	151	1	1
	Yes	32	43	1.6(1.0, 2.7)*	2.7(1.1, 3.0)*
Age	≤20 years(ref.)	17	19	1	1
	20–34 years	167	153	.8(.4,1.6)	.6(.3, 1.3)
	≥35 years	35	22	.5(.2,1.3)	.4(.1,.9)*
Education level	No formal education(ref.)	43	43	1	1
	Primary education	55	60	1.1(0.6, 1.9)	0.8(.4, 1.4)
	Sec. & preparatory	65	61	.8(.5,1.5)	.6(0.3,1.1))
	College and above	26	60	1.9(1.1, 3.5)	1.3(0.6,2.8)
Occupation	Employee	36	44	1.5(.9,2.4)	.9(0.5,1.8)
	Merchant	18	24	1.6(.8,3.1)	1.3(0.6,2.6)
	Housewife(ref.)	141	117	1	1
	Daily laborer	8	5	0.7(.2, 2.3)	0.6(0.2,1.9)
	Students	16	4	0.3(0.2, .9)*	0.3(0.1,1.8)

Statically significant at **P< 0.01 and *P< 0.05.

## Discussion

This study evaluated the health system responsiveness of maternity care in Hadiya Zone public hospitals from a user’s perspective. The findings indicated that 53.0% of users reported good responsiveness in delivery care, which is higher than the reported scores of 41.8% and 49.0% in the obstetrics and gynaecology departments of teaching hospitals in Mashhad and Tehran, Iran [29, 30]. This variation may be due to differences in the study setting, design, population, year of the study, and various training given in these study areas (respectful maternity care). Yet the finding was lower than the reported percentages in studies conducted in Iran and Germany: 58.4%, and 78% [31, 32]. This lower percentage might be due to social, economic or cultural differences since previous studies were conducted in upper middle- and high-income countries. Another reason may be that the COVID-19 pandemic affected some components of a responsive domain, specifically having a companion of choice during delivery (social support domain).

According to the results of this study, dignity was the best performing domain in maternity care. The same results were reported in studies in the Netherlands [25], Thailand [33] and Kenya [34], Iran [17, 31], and the Democratic Republic of Congo [35]. In contrast to this study, studies conducted in Iran [29, 30] and Ethiopia [36] reported that confidentiality was the best performing domain. A likely cause of high confidentiality is that these two studies were carried out using a sensitive issue that requires confidentiality rather than maternity care, which does not. For example, the study conducted in Ethiopia focused on HIV/AIDS treatment and care services, which may require a confidentiality domain more than dignity domains.

The domain with the lowest performance score in maternity care was the choice of health care provider. This finding is comparable to the findings of several other studies [17, 25, 29, 33, 34, 37–39]. In contrast, a study conducted in Tehran, indicated that the autonomy domain received the lowest score [31]. A potential reason for this low performance is that the study’s methodology was a household survey in which respondents may have paid more attention to the autonomy domain than to the choice of provider domain.

In this study, women aged ≥ 35 were 60% less probable to rate maternity care responsiveness as poor than those in the age range of ≤ 20 years. This finding is similar to that of a study conducted in Thailand [33], but it differs from that of a study in the obstetrics and gynaecology departments of teaching hospitals in Mashhad, Iran [30], in which older mothers rated responsiveness at lower levels. These lower levels may be due to older mothers having a poor understanding of their rights during maternity care.

Obstetrics complications during pregnancy were significantly associated with a poor rating of responsiveness in maternity care, a finding similar to that of a study in the Netherlands [25]. A probable explanation is that mothers with obstetrics complications expect an increased level of care; therefore, they give responsiveness a lower rating.Mothers who had undergone a caesarean delivery were 60% less likely to report poor responsiveness in maternity care compared to those who had a vaginal delivery. This finding is similar to that of a study conducted in the Democratic Republic of Congo [35]. A possible reason for this finding is that mothers undergo a caesarean section with medical justification based on both individual preferences and obstetrics complications. Similarly, a study conducted in the Netherlands found that Caesarean delivery was significantly associated with poor responsiveness performance [25].

Residing in an urban area was significantly associated with a poor rating of responsiveness in maternity care. A potential reason for the poor responsiveness is that urban women are more knowledgeable of their rights during maternity care and, for that reason, hold higher expectations [36]. Consequently, women unaware of their rights during delivery may report a greater satisfaction with responsiveness.

The strengths of this study include the fact that study participants were selected using the systematic sampling approach to ensure the representativeness of the study, and different approaches were used to maintain the quality of data. This study tried to assess non-medical medical care factors, which are the neglected aspects of healthcare services. All public hospitals in the Hadiya zone were included in the study and appropriate statistical methods were used to identify relations between the dependent and independent variables. The limitation of this study includes several factors. This study was not supported by a qualitative method of research into maternity care; therefore, it was not possible to determine the reasons from different perspectives for the poor responsiveness performance. The study assessed health system responses in maternity care, indirectly, by recording mothers’ views, which might either decrease or increase the performance level achieved. In addition, the study was conducted during the Covid-19 pandemic, which may have affected the responses to maternity care within the health system at this time. Finally, the study did not include maternity care staff and health facility issues in the research.

## Conclusion

In the hospitals under investigation, responsiveness in maternity care was found to be good. Variations occurred across all the domains; in particular, having a maternal age of ≥ 35 years, being urban, having obstetric complications during pregnancy, and vaginal delivery were all factors associated with poor health system responsiveness in maternity care. The findings of this study suggest that the ministry of health and regional health bureau needs to pay attention to health system responsiveness as an indicator of the quality of maternity care. Responsiveness indicators are an important tool to assess the performance of maternity care staff and the healthcare system. The Ministry of Health, Regional Health Bureau, hospital authorities and maternity care providers should pay attention to these non-medical aspects of care.

## Supporting information

S1 File Consent form, English and amharic questionnaire. (DOC)

S2 File SPSS. (SAV)

S1 Fig Diagrammatic presentation of the sampling technique. (TIF)

## Abbreviations

ANC: Antenatal care
AOR: Adjusted odd ratio
CI: Confidence interval
COR: crude odds ratio
NGO: Non–governmental organization
SPSS: statistical package for social sciences
WUNEMMTH: Wachemo University Nigist Eleni Mohammed Memorial Teaching Hospital
WHO: World Health Organisation
