# Supplementary material for: Health system responsiveness in maternity care at Hadiya zone public hospitals in Southern Ethiopia: Users’ perspectives
Source: PLoS One. 2021 Oct 14;16(10):e0258092. doi: 10.1371/journal.pone.0258092 (PMC8516277; doi:10.1371/journal.pone.0258092)
Supplement: S1 File — (DOC) [file pone.0258092.s001.doc]

# **7. Annexes**

**Annex I. Information and Informed Consent**

**Dear Respondent:**

My name is _____________________. This questionnaire is prepared to assess the Health System Responsiveness in Delivery care of Wachemo University Nigist Eleni Mohammed Memorial Teaching Hospital, Hadiya Zone, and Southern Ethiopia. You are selected and included in the study as part of the sample population to complete the questionnaire designed by the researcher. Since there is no research done on this topic in the study area, provision of such data on this very important subject may alert researchers, health workers, community and individuals for better health of mothers and newborns. It will be used as reference for those who are interested to perform a research on the same topic. The study will involve you completing the questionnaire that is enclosed with this letter and it will not take more than 20 minutes to complete. Confidentiality and anonymity is fully assured, as your name is not required on the questionnaire and only the research team will have access to the results.

**Informed Consent Form**

Dear my participant, I would be grateful if you would participate in this study by completing this questionnaire. It hoped that the findings of this study would help to identify factors associated to Health System Responsiveness in Delivery care of Wachemo University Nigist Eleni Mohammed Memorial Teaching Hospital, Hadiya Zone, and Southern Ethiopia. Be assured that the information you provide will be used for research purposes only and will be treated as confidential and participation will be based on voluntarily will**.** I also would like to assure you that any time you feel uncomfortable participating on the study and withdrawing from the study. The study does not have any effect on the service you obtain from the respective health facility.

If you would like to know more, please contact:

**Address of the Principal Investigators Name** Ritbano Ahmed, 0910143410

I thank you in advance for taking your time to answer questions.

Do you have any opinion regarding this study?

Do you agree to participate in this study?

Yes, continue Sign________ Date______ No, thank you!

Name of the data collector_____________________________ Sign________ Date______

Date_________________Start time_____________End time__________________

**Annex II: Data Collection Tool**

| **Part I – Questions related to socio- demographic characteristics of the study participants/** **ክፍል I - ከጥናቱ ተሳታፊዎች ማህበራዊና ስነ-ህዝብ ሁኔታ ጋር የተዛመዱ ጥያቄዎች** | | | |
| --- | --- | --- | --- |
| **No** | **Questions** | **Responses** | **Skip to** |
|  | Code/MRN | _________________ |  |
|  | How old are you? Age at interview in completed year | _________________ years |  |
|  | What is your current marital status? | 1. Single 2. Married 3. Other(specify)_____________ |  |
|  | What is the highest level of education you completed? | 1. No education 2. No formal education but read and write 3. Grade 1 to 8 4. Grade 9 to 12 5. Above grade 12 |  |
|  | What is your occupation? | 1. Government employee 2. Employee of private/NGO 3. Merchant 4. Housewife 5. Others(specify)________________ |  |
|  | What is your religion? | - 1. Protestant   2. Orthodox   3. Muslim   4. Catholic   5. Other(specify) ________________ |  |
|  | What is your ethnicity? | 1. Hadiya 2. Kambata 3. Silte 4. other(specify) ________________ |  |
|  | Residence | 1. Urban 2. Rural |  |
|  | Average family income per month (Eth. Birr} | - Mother_______________Birr/month - Husband __________birr/month - Additional income_________birr |  |
|  |  |  |  |

| **Part II: Questions related to obstetric history /** **ከወሊድ ጋር የተዛመዱ ጥያቄዎች** | | | | |
| --- | --- | --- | --- | --- |
| **No** | | **Questions** | **Responses** **የማህፀን ህክምና** | **Skip** |
|  | How many times you became pregnant? / | | _______________ |  |
|  | How many births have you given? | | _______________ |  |
|  | Have you had antenatal care follow up? | | - 1. No   2. Yes | If no, go to Q5 |
|  | If yes, number of visits | | ________ |  |
|  | Perinatal health care path? | | - 1. Start ANC with midwife, referred during ANC to Gynecologist   2. Start ANC with midwife, not referred   3. Start ANC with midwife, referred during labor to Gynecologist   4. No ANC, I came here during labor |  |
|  | Onset of labour | | - 1. Spontaneous   2. Induction   3. Elective Cd |  |
|  | What is route of current delivery? | | - 1. Vaginal   2. Caesarian delivery |  |
|  | Intervention during labor | | 1. No 2. Yes, no emergency intervention 3. Yes, emergency intervention |  |
|  | Antipain given during labor | | - 1. No requested   2. No medication received after requesting   3. Pain medication received after requesting |  |
|  | Time of delivery | | - 1. 0-8   2. 8-16   3. 16-24 |  |
|  | Day of delivery | | 1. Weekend 2. Work day |  |
|  | Length of hospital stay | | _____________ day(s) |  |
|  | Duration of labor pain | | _____________ hours |  |
|  | Who has conducted the delivery?(qualification) | | ___________________________ |  |
|  | Adverse outcome of child | | 1. No 2. Yes |  |
|  | Hospital admission of the mother/complication? | | - 1. No   2. Yes |  |
|  | Hospital admission of the mother? | | - 1. No   2. Yes |  |

**Part III: The eight domains with the questions developed for the maternity care**

| **General direction: Rate your experience from the choices at right for each question below.** | | | |
| --- | --- | --- | --- |
| **No** | **Domain and Items** | | **Rating** |
| **Dignity/** | | | |
|  | I was treated respectfully during my experience. | | - Strongly disagree - Disagree - Neutral - Agree - Strongly agree |
|  | The physical examination and treatments were done in a way that respected my privacy. | | 1. Strongly disagree 2. Disagree 3. Neutral 4. Agree 5. Strongly agree |
|  | I was encouraged to ask questions about diseases, treatments and care. | | 1. Strongly disagree 2. Disagree 3. Neutral 4. Agree 5. Strongly agree |
|  | The health care provider gave me personal attention during history taking, physical examination and treatment. | | 1. Strongly disagree 2. Disagree 3. Neutral 4. Agree 5. Strongly agree |
|  | I was encouraged to discuss my concerns freely. | | 1. Strongly disagree 2. Disagree 3. Neutral 4. Agree 5. Strongly agree |
| **Autonomy** | | | |
| 1. . | I was involved in making decisions for my treatment | | 1. Strongly disagree 2. Disagree 3. Neutral 4. Agree 5. Strongly agree |
|  | I received information about other types of treatments or tests. | | 1. Strongly disagree 2. Disagree 3. Neutral 4. Agree 5. Strongly agree |
|  | I had a choice to refuse examinations or treatments. | | 1. Strongly disagree 2. Disagree 3. Neutral 4. Agree 5. Strongly agree |
|  | I was asked for permission before starting testing or treatments. | | 1. Strongly disagree 2. Disagree 3. Neutral 4. Agree 5. Strongly agree |
| **Confidentiality** | | | |
|  | I was given the opportunity to speak privately with health care providers. | | 1. Strongly disagree 2. Disagree 3. Neutral 4. Agree 5. Strongly agree |
|  | My personal information was kept confidential. | | 1. Strongly disagree 2. Disagree 3. Neutral 4. Agree 5. Strongly agree |
|  | My medical record was kept confidential. | | 1. Strongly disagree 2. Disagree 3. Neutral 4. Agree 5. Strongly agree |
|  |  | |  |
| **Communication** | | | |
|  | Things were explained clearly by my health care provider in way I could understand. | | 1. Strongly disagree 2. Disagree 3. Neutral 4. Agree 5. Strongly agree |
|  | I was encouraged to ask questions about my health problems, treatments and care. | | 1. Strongly disagree 2. Disagree 3. Neutral 4. Agree 5. Strongly agree |
|  | I was given enough time to ask questions about my health problems and treatments. | | 1. Strongly disagree 2. Disagree 3. Neutral 4. Agree 5. Strongly agree |
|  | My health care providers were responsive to my questions. | | 1. Strongly disagree 2. Disagree 3. Neutral 4. Agree 5. Strongly agree |
|  | My health care providers listened carefully to me. | | 1. Strongly disagree 2. Disagree 3. Neutral 4. Agree 5. Strongly agree |
| **Prompt attention** | | | |
|  | I received prompt attention at this health facility. | | 1. Strongly disagree 2. Disagree 3. Neutral 4. Agree 5. Strongly agree |
|  | The amount of time I waited before being attended to was acceptable. | | 1. Strongly disagree 2. Disagree 3. Neutral 4. Agree 5. Strongly agree |
|  | The travel time to this health service was acceptable. | | 1. Strongly disagree 2. Disagree 3. Neutral 4. Agree 5. Strongly agree |
| **Social support** | | | |
|  | It was easy to have family and friends visit me. | | 1. Strongly disagree 2. Disagree 3. Neutral 4. Agree 5. Strongly agree |
|  | I had the option of having an attendant stay with me during various stages of diagnosis and treatment. (This may not apply in special cases.) | | 1. Strongly disagree 2. Disagree 3. Neutral 4. Agree 5. Strongly agree |
| 1. . | My family and friends were able to bring care and services, such as food or other consumables | | 1. Strongly disagree 2. Disagree 3. Neutral 4. Agree 5. Strongly agree |
|  |  | |  |
| **Choice** | | | |
|  | I had freedom in choosing this health care provider. | | 1. Strongly disagree 2. Disagree 3. Neutral 4. Agree 5. Strongly agree |
|  | I had the option to choose health care services I do not normally use. | | 1. Strongly disagree 2. Disagree 3. Neutral 4. Agree 5. Strongly agree |
|  | Continuity of care was provided by my individual health care provider. | | 1. Strongly disagree 2. Disagree 3. Neutral 4. Agree 5. Strongly agree |
|  |  | |  |
| **Quality of basic amenities** | | | |
|  | | | |
|  | This health care facility, including toilets, examination room and linen, was clean. | | 1. Strongly disagree 2. Disagree 3. Neutral 4. Agree 5. Strongly agree |
|  | The amount of space provided was adequate. | | 1. Strongly disagree 2. Disagree 3. Neutral 4. Agree 5. Strongly agree |
|  | Health care personnel practised good hygiene (hands, clothes, linens, etc.). | | 1. Strongly disagree 2. Disagree 3. Neutral 4. Agree 5. Strongly agree |
|  | The department, bedroom, and bathroom were clean. | | 1. Strongly disagree 2. Disagree 3. Neutral 4. Agree 5. Strongly agree |
|  | Waiting areas and rooms had good air quality and ventilation. | | 1. Strongly disagree 2. Disagree 3. Neutral 4. Agree 5. Strongly agree |
| **Importance of Domains** | | | |
|  | Write the most important domains in delivery care | ________________________ | |

**Thank you for your participation!!**

**ዋቸሞ ዩኒቨርሲቲ ህክምናና ጤና ሳይንስ ኮሌጅ**

**ሚድዋይፈሪ ጤና ትምህርት ክፍል**

**አማርኛ መጠይቅ**

**አባሪ II. የመረጃና የስምምነት ዉል ቅፅ**

**የምርምሩ/የጥናቱ ርዕስ**፡- በደቡብ ኢትዮጵያ በሃዲያ ዞን ሆስፒታሎች ውስጥ ሲወለድ የጤና ስርዓት ምላሽ ሰጪነት የተጠቃሚዎች አመለካካትበደቡብ ኢትዮጵያ በሃዲያ ዞን ሆስፒታሎች ውስጥ የእናቶች እንክብካቤ የጤና ስርዓት ምላሽ-የተጠቃሚዎች አመለካካትን ፡፡

ጤና ይስጥልን! ---------------------------------------- እባላለሁ፡፡ የምሰራዉ በዋቸሞ ዩኒቨርሲቲ ዩኒቨረሲቲ ሚድዋይፈሪ ጤና ትምህርት ክፍል ምርምር በማድረግ ላይ ከሚገኙት መምህር ሪጥባኖ አህመድ ጥናት አድራጊ ጋር ሲሆን፤ በደቡብ ኢትዮጵያ በሃዲያ ዞን ሆስፒታሎች ውስጥ የእናቶች እንክብካቤ የጤና ስርዓት ምላሽ ምላሽ-የተጠቃሚዎች አመለካካትን በተመለከተ ለማወቅ ቃለ መጠይቅ ቅፅ እያስሞለሁኝ ሲሆን **የጥናቱ ዓላማ** በደቡብ ኢትዮጵያ በሃዲያ ዞን ሆስፒታሎች ውስጥ ሲወለድ የጤና ስርዓት ምላሽ ሰጪነት የተጠቃሚዎች አመለካካት ሁኔታን ምን ደረጃ ላይ እንዳለው ለማወቅና ከዚህ ጋር ተያያዥነት ያላቸውን ዋና ዋና ጉዳዮችን ለመለየት ነው፡፡ጥያቄዎቹን ለመጠየቅ 20-25ደቂቃ ሊፈጅ ይችላል፡፡ በጥናቱ ላይ የእርሶ ስምና አድራሻ አይጠቀስም፡፡ የሚሰጡትም መረጃ ከዚህ ጥናት አላማ ውጭ ለሌላ አካል ተላልፎ አይሰጥም ሚስጥራዊነቱም የተጠበቀ ነው፡፡ በዚህ ጥናት ላይ በመሳተፎት የሚደርስቦት ጉዳት ወይም የተለየ ጥቅም አይኖርም፡፡በዚህ ጥናት መሳተፍ ፈቃደኛ ካልሆኑ፤ በመጠይቁ መሀል ማቋረጥ ከፈለጉ ወይንም መመለስ የማይፈልጉት ጥያቄ ሲኖር የማቁዋረጥ ሙሉ መብት እንዳሎት ልገልጽሎት እወዳለሁ፡፡ በጥናቱ ላይ ለመሳተፍ የእርሶ ትብብር እና ፈቃደኝነት በጉዳዩ ላይ የሚነሱ ችግሮችን ለመለየት እጅግ ጠቃሚ ስለሆነ በጥናቱ ላይ በፍቃደኝነት እንዲሳተፉ በትህትና እንጠይቃለን፡፡ከላይ በተሰጠኝ መረጃ መሰረት በዚህ ጥናት ላይ ለመሳተፍ ፍቃደኛ ነኝ፡፡

ፊርማ ----------------------

**ጥናቱን የሚያካሂደዉ ሰዉ አድራሻ፡** ስም፡ ሪጥባኖ አህመድ ስ.ቁ ፡ 0910143710

የመረጃሰብሳቢው ስም ------------------------------- ፊርማ-------------------------

ቃለ መጠይቅ የተደረገበት ቀን----------------------

**አባሪ II: የመረጃ አሰባሰብ መሣሪያ**

በደቡብ ኢትዮጵያ በሃዲያ ዞን ሆስፒታሎች ውስጥ የእናቶች እንክብካቤ የጤና ስርዓት ምላሽ የተጠቃሚዎች አመለካካትን በተመለከተ ለማወቅ የተዘጋጀ ቃለ መጠይቅ ቅፅ::

| **ክፍል አንድ : ማህበራዊና ኢኮኖሚያዊ ሁኔታ መጠይቆች** | | | | | | | | | | |
| --- | --- | --- | --- | --- | --- | --- | --- | --- | --- | --- |
| **ተ.ቁ** | **ጥያቄ** | | | **መልስ** | | | | | | |
| **100** | ቁጥር//MRN | | | __________ | | | | | | |
| **101** | እድሜዎ ስንት ነው? | | | __________ **አመት** | | | | | | |
| **102** | የአሁኑ የጋብቻ ሁኔታዎ ምንድ ነው? | 1. ያገባች | | | | 1. ያለገባች | | 1. የፈታች | | 1. ባል የሞተባት |
| **103** | የት/ት ደረጃዎ ? | 1. ያልተማረች 2. መፃፍና ማንበብ የምትችል 3. አንደኛ ደረጃ ያጠናቀቀ. | | | | | | 1. 2ኛ ደረጃ ያጠናቀቀች 2. 12 ኛ ክፍል ያጠናቀቀ 3. ኮሌጅና ከዚያ በላይ | | |
| **104** | ሥራህ ምንድን ነው? | 1. የመንግስት ተቀጣሪ 2. መንግስታዊ ያልሆነ ድርጅት 3. ነጋዴ | | | | | | 1. የቤት እመቤት 2. የቀን ሰራተኛ 3. ተማሪ 4. ሌሎች (ያብራሩ) | | |
| **105** | ሃይማኖትዎ ምንድን ነው ? | | | 1. ፕሮቴስታንት 2. ኦርቶዶክስ | | | | 1. ሙስሊም 2. ሌሎች(ያብራሩ) ------- | | |
| **106** | ብሄርዎ ምንድን ነው? | | | 1. ሃዲያ 2. ከምባታ 3. ስልጤ | | | | 1. ጉራጌ 2. አማራ 3. ሌላ ካለ ይገለጽ_______ | | |
| **107** | መኖሪያ? | | | 1. ገጠር | | | | 1. ከተማ | | |
| **108** | በወር አማካይ የቤተሰብ ገቢ (ኢት. ብር) | | | _______________ ብር / በወር | | | | | | |
| **ክፍል ሁለት፡- የስነተዋልዶ ታሪክን በተመለከተ/የወሊድሁኔታ** | | | | | | | | | | |
| **200** | እስካሁን ያለዉ የእርግዝናና ወሊድ ብዛት? | | | 1. የእርግዝና ብዛት ________ 2. የወሊድ ብዛት ________ 3. የዉርጃ ብዛት _______ 4. በህይወት ያሉ ልጆች ብዛት ________ | | | | | | |
| **201** | የእርግዝና ክትትል ነበረሽ? | | | 0. የለም 1. አዎ | | | የለም ከሆነ ወደ ጥያቄ ተቁ. 203 ይሻገሩ | | | |
| **202** | ለጥ. ቁ 201 አዎ ከሆነ ፣ የጉብኝቶች ብዛት? | | | ________ | | | | | | |
| **203** | የምጥ አጀማመር ሁኔታ? | | | 1. በራሱ ጊዜ 2. በምጥ መርፌ 3. ተመራጭ ቀዶ ጥገና | | | | | | |
| **204** | የአሁን የወለድሽበት መንገድ ምንድነው? | | | 1.በማህጸን 2. በቀዶ ጥገና | | | | | | |
| **205** | በምጥ ጊዜ ጣልቃገብነት | | | 1. የለም 2. አዎ ፣ ድንገተኛ ጣልቃ ገብነት የለም 3. አዎ ፣ የድንገተኛ ጊዜ ጣልቃ ገብነት | | | | | | |
| **206** | በምጥ ጊዜ ህመም ማስታገሻ መድሃኒት ተሰጥቶሻል | | | 1. አልተጠየቀም 2. ከጠየቁ በኋላ ምንም መድሃኒት አልተገኘለትም 3. ከጠየቁ በኋላ የህመም ማስታገሻ መድሃኒት ተቀብየለሁ | | | | | | |
| **207** | ስንት ሰዓት ተገላገልሽ? | | | ቀን ________ሰዓት  ማታ________ሰዓት | | | | | | |
| **208** | የወለድሽው ቀን? | | | 1. የሳምንት እረፍት ቀን  2. የስራ ቀን | | | | | | |
| **209** | ሆስፕታል ምን ያህል ጊዜ ቆየሽ? | | | ________ቀን/ ________ሰዓት | | | | |  | |
| **210** | በምጥ ስንት ሰኣት ቆየሽ? | | | ________ሰዓት | | | | |  | |
| **211** | ማን አዋለደሽ?(ሙያ) | | |  | | | | |  | |
| **212** | የልጅሽ የጤና ሁኔታ ችግር ነበር? | | | 0. የለም 1. አዎ | | | | |  | |
| **213** | በ እርግዝና /በምጥ ምክንያት ሆስፕታል ተኝተሸ ነበር ? | | | 0. የለም 1. አዎ | | | | |  | |
| **214** | በህመም ምክንያት ልጅሽን ሆስፕታል አስተኝተሻል? | | | 0. የለም 1. አዎ | | | | |  | |
| **215** | የእርግዝና ክትትል ሁኔታ/ መንገድ እንዴት ነበር? | | 1. እዚው ጀምርኩ ፣ ለምጥ እዚህ መጠሁኝ :: 2. በሚድዋይፍ(ሌላ ቦታ) የእርግዝና ክትትል ጀምሬ ለቀሩት ክትትል ወደ ማህፀን ሐኪም(ሆስፕታል) ተላኩ :: 3. ሚድዋይፍ((ሌላ ቦታ) ጋር የቅድመ የወሊድ እንክብካቤን ጀምሬ, በምጥ ጊዜ ወደ ሐኪም(ሆስፕታል) ተላኩ :: 4. ከማህፀን ሐኪም ጋር ቅድመ ወሊድ እና የወሊድ እንክብካቤ አገኘሁኝ :: | | | | | | | |
| **ክፍል 3: ስምንት ጎራዎችን የያዘ ለእናቶች እንክብካቤ የሚውል የጤና ስርዓት ምላሽ መጠይቅ** | | | | | | | | | | |
| **ተ.ቁ** | **ጎራዎች እና ጥያቄዎች** | | | | **ደረጃ መስጠት** | | | | | |
| **1.ክብር/Dignity** | | | | | | | | | | |
| **መመሪያ: ከዚህ በታች ላለው ለእያንዳንዱ ጥያቄ በቀኝ በኩል ካሉት ምርጫዎች ተሞክሮዎን ይመዝኑ ፡፡** | | | | | | | | | | |
| 300 | በምጥ ወቅት በአክብሮት ተይዣለሁ ፡፡ | | | | 1. በጣም አልስማማም 2. አልስማማም 3. ገለልተኛ. | | | | 1. እስማማለሁ 2. በጣም እስማማለሁ | |
| 301 | አካላዊ ምርመራው እና ሕክምናው የእኔን ግላዊነት በሚከበረ መንገድ ተደረገ ፡፡ | | | | 1. በጣም አልስማማም 2. አልስማማም 3. ገለልተኛ. | | | | 1. እስማማለሁ 2. በጣም እስማማለሁ | |
| 302 | ስለ ምጥ ፣ ህክምና እና እንክብካቤ ጥያቄዎችን እንድጠይቅ ተበረታቼ ነበር ፡፡ | | | | 1. በጣም አልስማማም 2. አልስማማም 3. ገለልተኛ. | | | | 1. እስማማለሁ 2. በጣም እስማማለሁ | |
| 303 | በታካሚነት ጊዜ ፣ ​​የአካል ምርመራ እና ህክምና ወቅት የጤና ባለሙያው የግል ትኩረት ሰጠኝ ፡፡ | | | | 1. በጣም አልስማማም 2. አልስማማም 3. ገለልተኛ. | | | | 1. እስማማለሁ 2. በጣም እስማማለሁ | |
| 304 | የእኔን ጭንቀቶች በነጻ እንድወያይ ማበረታቻ ተደረገልኝ ፡፡ | | | | 1. በጣም አልስማማም 2. አልስማማም 3. ገለልተኛ. | | | | 1. እስማማለሁ 2. በጣም እስማማለሁ | |
| **2.ራስን በራስ ማስተዳደር/ Autonomy** | | | | | | | | | | |
| 305 | በሕክምናዬ ውሳኔዎች ውስጥ ተሳትፌ ነበር:: | | | | 1. በጣም አልስማማም 2. አልስማማም 3. ገለልተኛ. | | | | 1. እስማማለሁ 2. በጣም እስማማለሁ | |
| 306 | ስለ ሌሎች የሕክምና ዓይነቶች ወይም ምርመራዎች መረጃ ደርሶኛል ፡፡ | | | | 1. በጣም አልስማማም 2. አልስማማም 3. ገለልተኛ. | | | | 1. እስማማለሁ 2. በጣም እስማማለሁ | |
| 307 | ምርመራዎችን ወይም ሕክምናዎችን ላለመቀበል ምርጫ ነበረኝ ፡፡ | | | | 1. በጣም አልስማማም 2. አልስማማም 3. ገለልተኛ. | | | | 1. እስማማለሁ 2. በጣም እስማማለሁ | |
| 308 | ምርመራ ወይም ሕክምና ከመጀመርዎ በፊት ፈቃድ ተጠይቄያለሁ ፡፡ | | | | 1. በጣም አልስማማም 2. አልስማማም 3. ገለልተኛ. | | | | 1. እስማማለሁ 2. በጣም እስማማለሁ | |
| **3.ምስጢራዊነት/ Confidentiality** | | | | | | | | |  | |
| 309 | ከጤና እንክብካቤ አቅራቢዎች ጋር በግል ለመነጋገር እድሉ ተሰጠኝ ፡፡ | | | | 1. በጣም አልስማማም 2. አልስማማም 3. ገለልተኛ. | | | | 1. እስማማለሁ 2. በጣም እስማማለሁ | |
| 310 | የግል መረጃዬ በሚስጥር ተጠብቆ ነበር ፡፡ | | | | 1. በጣም አልስማማም 2. አልስማማም 3. ገለልተኛ. | | | | 1. እስማማለሁ 2. በጣም እስማማለሁ | |
| 311 | የእኔ የሕክምና መዝገብ በሚስጥር ተጠብቆ ቆይቷል ፡፡ | | | | 1. በጣም አልስማማም 2. አልስማማም 3. ገለልተኛ. | | | | 1. እስማማለሁ 2. በጣም እስማማለሁ | |
| **4.መግባባት/Communication** | | | | | | | | | | |
| 312 | ለመረዳት የምችልበት መንገድ ነገሮች በጤና እንክብካቤ አቅራቢዬ ውስጥ በግልፅ ተብራርተዋል ፡፡ | | | | 1. በጣም አልስማማም 2. አልስማማም 3. ገለልተኛ. | | | | 1. እስማማለሁ 2. በጣም እስማማለሁ | |
| 313 | ስለጤንነቴ ችግሮች ፣ ሕክምናዎች እና እንክብካቤዎች ጥያቄዎችን እንድጠይቅ ተበረታቼ ነበር ፡፡ | | | | 1. በጣም አልስማማም 2. አልስማማም 3. ገለልተኛ. | | | | 1. እስማማለሁ 2. በጣም እስማማለሁ | |
| 314 | ስለጤንነቴ ችግሮችና ሕክምናዎች ጥያቄዎችን ለመጠየቅ በቂ ጊዜ ተሰጠኝ ፡፡ | | | | 1. በጣም አልስማማም 2. አልስማማም 3. ገለልተኛ. | | | | 1. እስማማለሁ 2. በጣም እስማማለሁ | |
| 315 | የእኔ የጤና እንክብካቤ ሰጭዎች ለጥያቄዎቼ መልስ ሰጡ ፡፡ | | | | 1. በጣም አልስማማም 2. አልስማማም 3. ገለልተኛ. | | | | 1. እስማማለሁ 2. በጣም እስማማለሁ | |
| 316 | የጤና እንክብካቤ ሰጭዎቼ በጥንቃቄ ያዳምጡኝ ነበር ፡፡ | | | | 1. በጣም አልስማማም 2. አልስማማም 3. ገለልተኛ. | | | | 1. እስማማለሁ 2. በጣም እስማማለሁ | |
| **5. አፋጣኝ ትኩረት/ Prompt attention** | | | | | | | | |  | |
| 318 | በዚህ የጤና ተቋም ውስጥ ፈጣን ትኩረት አግኝቻለሁ ፡፡ | | | | 1. በጣም አልስማማም 2. አልስማማም 3. ገለልተኛ. | | | | 1. እስማማለሁ 2. በጣም እስማማለሁ | |
| 319 | እንክብካቤ ከመገኘቴ በፊት የጠበቅኩት የጊዜ መጠን ተቀባይነት ነበረው ፡፡ | | | | 1. በጣም አልስማማም 2. አልስማማም 3. ገለልተኛ. | | | | 1. እስማማለሁ 2. በጣም እስማማለሁ | |
| 320 | ወደዚህ የጤና አገልግሎት የሚደረገው የጉዞ ጊዜ ተቀባይነት ነበረው ፡፡ | | | | 1. በጣም አልስማማም 2. አልስማማም 3. ገለልተኛ. | | | | 1. እስማማለሁ 2. በጣም እስማማለሁ | |
| **6.ማህበራዊ ድጋፍ/ Social support** | | | | | | | | | | |
| 321 | ቤተሰቦቼና ጓደኞቼ እኔን በቀላሉ ልጎቦኙ ችለዋል ፡፡ | | | | 1. በጣም አልስማማም 2. አልስማማም 3. ገለልተኛ. | | | | 1. እስማማለሁ 2. በጣም እስማማለሁ | |
| 322 | በምርመራ እና በሕክምና የተለያዩ ደረጃዎች ወቅት አንድ አገልጋይ ከእኔ ጋር የመቆየት አማራጭ ነበረኝ ፡፡(ይህ በልዩ ጉዳዮች ላይ ተፈፃሚ ላይሆን ይችላል) | | | | 1. በጣም አልስማማም 2. አልስማማም 3. ገለልተኛ. | | | | 1. እስማማለሁ 2. በጣም እስማማለሁ | |
| 323 | ቤተሰቦቼና ጓደኞቼ እንደ ምግብ ወይም ሌሎች ፍጆታዎችን የመሳሰሉ እንክብካቤዎችን እና አገልግሎቶችን ማቅረብ ችለው ነበር፡፡ | | | | 1. በጣም አልስማማም 2. አልስማማም 3. ገለልተኛ. | | | | 1. እስማማለሁ 2. በጣም እስማማለሁ | |
| **7. ምርጫ/Choice** | | | | | | | | | | |
| 324 | የጤና ባለሙያ የመምረጥ ነፃነት ነበረኝ ፡፡ | | | | 1. በጣም አልስማማም 2. አልስማማም 3. ገለልተኛ. | | | | 1. እስማማለሁ 2. በጣም እስማማለሁ | |
| 325 | በተለምዶ የማውቃቸውን የጤና እንክብካቤ አገልግሎቶች ለመምረጥ አማራጭ ነበረኝ ፡፡ | | | | 1. በጣም አልስማማም 2. አልስማማም 3. ገለልተኛ. | | | | 1. እስማማለሁ 2. በጣም እስማማለሁ | |
| 326 | በአንድ ጤና ባለሙያ እንክብካቤ ቀጣይነት የተሰጠው ነው ፡፡ | | | | 1. በጣም አልስማማም 2. አልስማማም 3. ገለልተኛ. | | | | 1. እስማማለሁ 2. በጣም እስማማለሁ | |
| **8. የመሠረታዊ መገልገያዎች ጥራት/ Quality of basic amenities** | | | | | | | | | | |
| 327 | መጸዳጃ ቤቶች ፣ የምርመራ ክፍሉ እና አንሶላዎችን ጨምሮ ይህ የጤና እንክብካቤ ተቋም ንፁህ ነበር ፡፡ | | | | 1. በጣም አልስማማም 2. አልስማማም 3. ገለልተኛ. | | | | 1. እስማማለሁ 2. በጣም እስማማለሁ | |
| 338 | የተሰጠው ቦታ በቂ ነበር ፡፡ | | | | 1. በጣም አልስማማም 2. አልስማማም 3. ገለልተኛ. | | | | 1. እስማማለሁ 2. በጣም እስማማለሁ | |
| 339 | የጤና አጠባበቅ ሠራተኞች ጥሩ ንፅህናን (እጆችን ፣ ልብሶችን ፣ መከለያዎችን ፣ ወዘተ ...) ያካሂዱ ነበር ፡፡ | | | | 1. በጣም አልስማማም 2. አልስማማም 3. ገለልተኛ. | | | | 1. እስማማለሁ 2. በጣም እስማማለሁ | |
| 330 | ክፍሉ ፣ መኝታ ቤቱና መታጠቢያ ቤቱ ንፁህ ነበር ፡፡ | | | | 1. በጣም አልስማማም 2. አልስማማም 3. ገለልተኛ. | | | | 1. እስማማለሁ 2. በጣም እስማማለሁ | |
| 331 | ማቆያ ቦታ እና ክፍሎች ጥሩ የአየር ጥራት እና አየር ነበረው ፡፡ | | | | 1. በጣም አልስማማም 2. አልስማማም 3. ገለልተኛ. | | | | 1. እስማማለሁ 2. በጣም እስማማለሁ | |
| **ክፍል 4: የጎራዎች አስፈላግነት** | | | | | | | | | | |
| 1 | የትኛው ጎራ ነው በወሊድ ወቀት በጣም አስፈላጊ? | | | | ________________________ | | | | | |

**ለተሳትፎዎ እናመሰግናለን!!**
