# Supplementary figures and images for: Health system responsiveness in maternity care at Hadiya zone public hospitals in Southern Ethiopia: Users’ perspectives
Source: PLoS One. 2021 Oct 14;16(10):e0258092. doi: 10.1371/journal.pone.0258092 (PMC8516277; doi:10.1371/journal.pone.0258092)

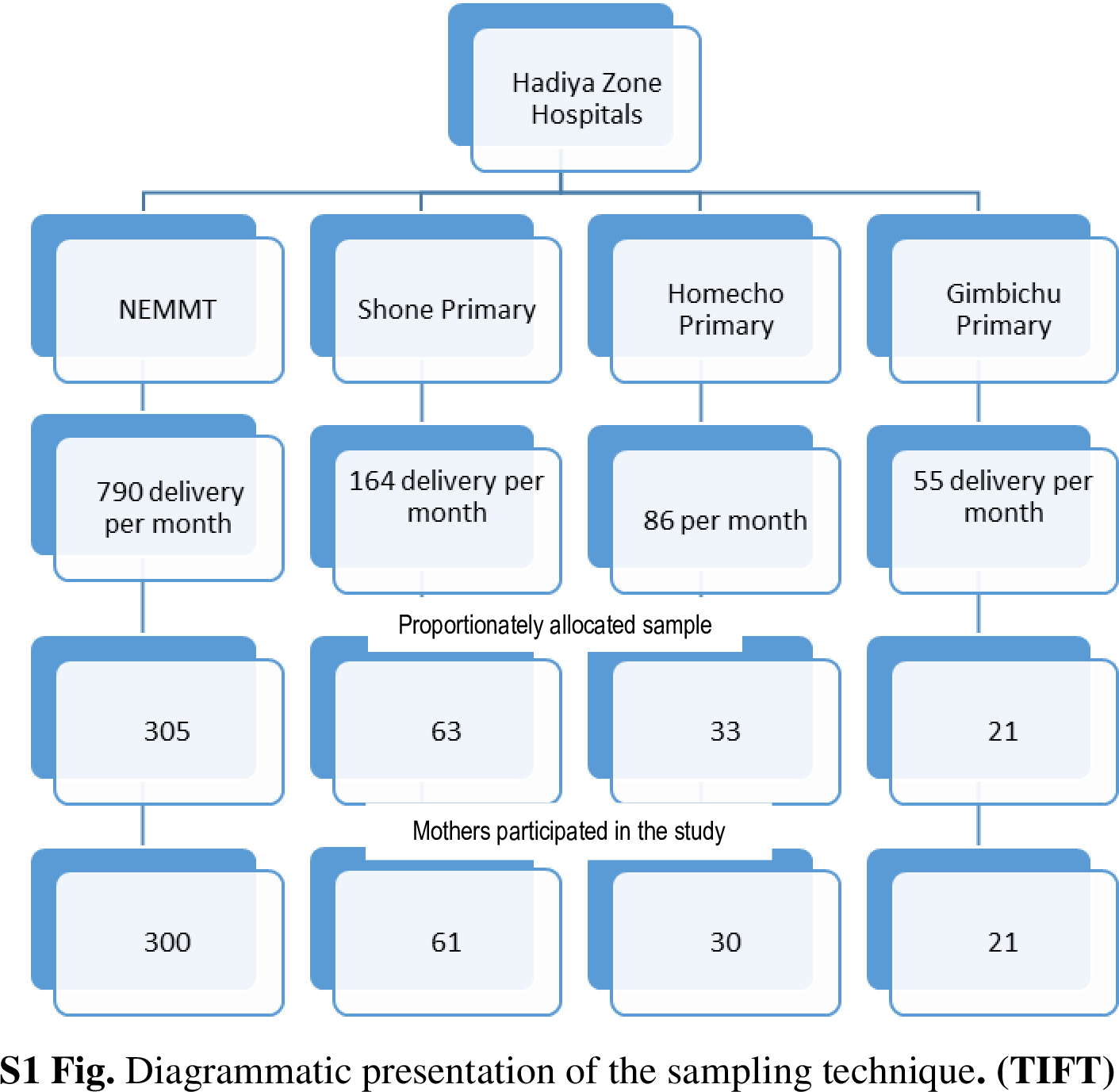

Supplement: S1 Fig — (TIF) [file pone.0258092.s003.tif]
